# High‐fat diet protects the blood–brain barrier in an Alzheimer's disease mouse model

**DOI:** 10.1111/acel.12818

**Published:** 2018-08-06

**Authors:** Shirin Elhaik Goldman, David Goez, David Last, Sharone Naor, Sigal Liraz Zaltsman, Inbal Sharvit‐Ginon, Dana Atrakchi‐Baranes, Chen Shemesh, Rachel Twitto‐Greenberg, Shoval Tsach, Roni Lotan, Alicia Leikin‐Frenkel, Aviv Shish, Yael Mardor, Michal Schnaider Beeri, Itzik Cooper

**Affiliations:** ^1^ The Joseph Sagol Neuroscience Center, Sheba Medical Center Tel Hashomer Ramat Gan Israel; ^2^ Gonda Brain Research Center Bar Ilan University Ramat‐Gan Israel; ^3^ The Advanced Technology Center, Sheba Medical Center Tel‐Hashomer Ramat‐Gan Israel; ^4^ Pharmacology Division, Faculty of Medicine, The Institute for Drug Research, School of Pharmacy Hebrew University of Jerusalem Jerusalem Israel; ^5^ Department of Psychology Bar Ilan University Ramat‐Gan Israel; ^6^ The Bert W. Strassburger Lipid Center, Sheba Medical Center Tel‐Hashomer Ramat‐Gan Israel; ^7^ Sackler Faculty of Medicine Tel‐Aviv University Tel‐Aviv Israel; ^8^ Department of Psychiatry The Icahn School of Medicine at Mount Sinai New York New York; ^9^ The Interdisciplinary Center Herzliya Israel

**Keywords:** Alzheimer’s disease, amyloid beta, blood–brain barrier, cholesterol, high‐fat diet, insulin resistance, MRI, Tg2576 mice

## Abstract

Type 2 diabetes (T2D) is associated with increased risk of Alzheimer's disease (AD). There is evidence for impaired blood–brain barrier (BBB) in both diseases, but its role in the interplay between them is not clear. Here, we investigated the effects of high‐fat diet (HFD), a model for T2D, on the Tg2576 mouse model of AD, in regard to BBB function. We showed that HFD mice had higher weight, more insulin resistance, and higher serum HDL cholesterol levels, primarily in Tg2576 mice, which also had higher brain lipids content. In terms of behavior, Tg2576 HFD mice were less active and more anxious, but had better learning in the Morris Water Maze compared to Tg2576 on regular diet. HFD had no effect on the level of amyloid beta 1–42 in the cortex of Tg2576 mice, but increased the transcription level of insulin receptor in the hippocampus. Tg2576 mice on regular diet demonstrated more BBB disruption at 8 and 12 months accompanied by larger lateral ventricles volume in contrast to Tg2576 HFD mice, whose BBB leakage and ventricular volume were similar to wild‐type (WT) mice. Our results suggest that in AD, HFD may promote better cognitive function through improvements of BBB function and of brain atrophy but not of amyloid beta levels. Lipid metabolism in the CNS and peripheral tissues and brain insulin signaling may underlie this protection.

## 1 INTRODUCTION

Alzheimer's disease (AD) is the most common form of neurodegenerative dementia accounting for 50%–80% of all age‐related dementia. AD is characterized by synaptic loss, neurodegeneration, and impairment of cognitive function. In 2016, there were approximately 5.4 million persons with AD in the US population. By 2050, this number is expected to increase by almost threefold, to 13.2 million (Hebert, Scherr, Bienias, Bennett, & Evans, 2003).

Type II diabetes (T2D) has been consistently associated with increased risk of cognitive decline (Arvanitakis, Wilson, Bienias, Evans, & Bennett, 2004), mild cognitive impairment (MCI) (Luchsinger et al., 2007) and dementia (Schnaider Beeri et al., 2004), both vascular dementia and AD (Peila, Rodriguez, & Launer, 2002). Of the shared features of AD and T2D, a major contributing factor to AD etiology is insulin resistance. Peripheral insulin resistance, even without T2D, is a risk factor and a common feature in AD (Janson et al., 2004; Talbot et al., 2012) and is associated with lower hippocampal volume and cerebral blood flow and poorer cognitive performance (Talbot et al., 2012). Peripheral insulin resistance could promote AD onset by reducing brain insulin uptake and by raising brain levels of Aβ, tau phosphorylation, oxidative stress, proinflammatory cytokines, advanced glycation end products, dyslipidemia, and apoptosis (Talbot et al., 2012). However, limited understanding of the vascular tangent between the two diseases hampers the development of therapeutics and preventive strategies against AD. The blood–brain barrier (BBB) hinders the entry of most molecules into the brain and enables active transportation of penetrated molecules to the brain (Zlokovic, 2011). Dysfunction of the BBB has been demonstrated in the pathogenesis and progression of AD (Kalaria, 2010), even before dementia onset (Skoog et al., 1998) and also in the aging human hippocampus, which worsens with mci, a condition preceding AD (Montagne et al., 2015). Changes in plasma glucose levels have been associated with altered BBB transport functions, with paracellular integrity (tight junction disruption) and with oxidative stress in the CNS microcapillaries (Prasad, Sajja, Naik, & Cucullo, 2014). At what stage BBB breakdown occurs in the brain, and whether it triggers the development of AD, remains, however, controversial.

High‐fat diet (HFD) is a well‐established model for generating impaired glucose tolerance and insulin resistance (Winzell & Ahren, 2004). The effect of HFD on the BBB is not well understood; data are limited, and the techniques to measure BBB functionality vary. Most studies support the notion that HFD increases BBB permeability. In some, HFD‐induced changes in BBB permeability were evident by increased leakage of Evans blue dye in 16‐week‐old mice brain (Nerurkar et al., 2011). In others, obesity exacerbated early postischemic BBB disruption in 16‐week‐old mice fed HFD. Tucsek et al. (2014) showed that 7 months of HFD did not change the BBB permeability (measured by IgG levels) in the hippocampus, but an exacerbated damage of the BBB was found at 24 months with HFD. The impact of HFD on memory and cognition is also controversial and depends on the composition of the fat and the time of exposure to HFD. Some experimental studies support the notion that HFD‐induced obesity exacerbates cerebral pathological alterations and the accompanying cognitive deficit in APP transgenic mice (Ho et al., 2004) while other claim for no effect (Kesby et al., 2015) or even improvement of memory in mice (Coscina, Yehuda, Dixon, Kish, & Leprohon‐Greenwood, 1986).

In this study, we investigated the effect of HFD on cognition and BBB function in Tg2576 mice, a transgenic mouse model of AD. Our study was conducted over 12 months in which BBB permeability was assessed in living mice using a novel technique based on delayed‐contrast MRI enabling detection of subtle BBB disruption (Zach et al., 2015). The results obtained suggest a protective effect induced by HFD on learning of AD‐like mice, through a mechanism that involved better barrier properties and brain morphology (normal ventricle volume), higher insulin receptor RNA expression in the hippocampus, and higher HDL cholesterol, but not through reduction in amyloid beta 1–42 (Aβ_1‐42_) levels.

## 2 RESULTS

### 2.1 Experimental design

A flowchart describing the experimental design is presented in Supporting Information Figure S1.

### 2.2 Tg mice gained more weight than WT mice fed with HFD

High‐fat diet significantly increased the weight of both WT and Tg mice over time (Supporting Information Figure S2). There was an interaction of genotype with HFD status (Supporting Information Figure S2A) such that the percentage of body weight gain was larger in the Tg HFD mice (Supporting Information Figure S2B, 168.5% ± 13.5) than in the WT CTRL mice (Supporting Information Figure S2B, 141.2% ± 26.4) while the Tg CTRL mice gained less weight (Supporting Information Figure S2B, 103.4% ± 12.5) compared to the WT CTRL mice (Supporting Information Figure S2B, 124% ± 10.18). These results demonstrate that body weight significantly increased over time due to HFD and that this increase was significantly larger for Tg mice.

### 2.3 Effects of HFD on insulin resistance and lipid content

**Figure 1:**
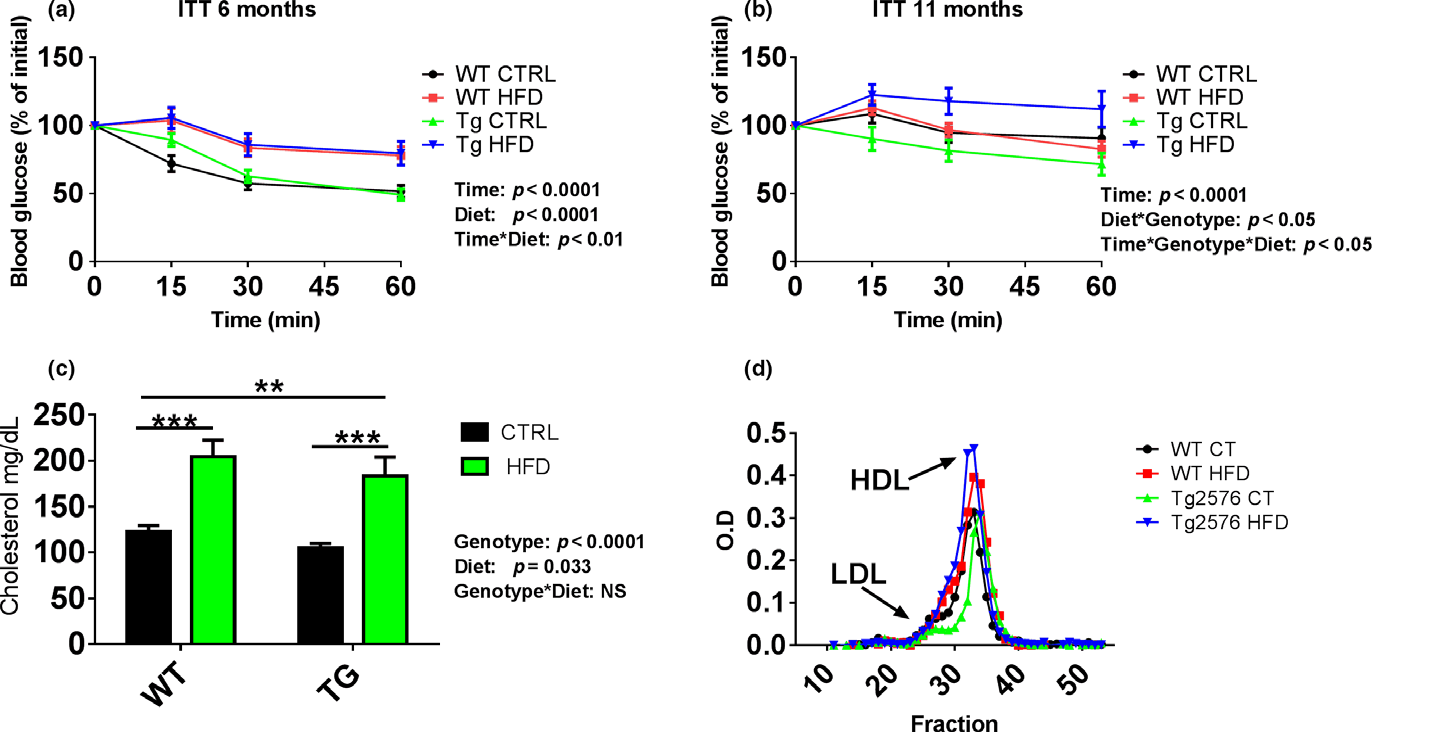
The effect of HFD on insulin resistance and serum cholesterol levels. ITT was performed at the age of 6 months (a) and 11 months (b). Blood glucose level (mg/dl) was measured 0, 15, 30, and 60 min following insulin injection. Values presented as the mean percentages ± *SEM* (*n* = 11–20). Tolerance is shown when there is a plateau in the graph. (a) At 6 months, HFD effect on insulin tolerance over time was significant (*p* < 0.01), that is, mice on HFD had consistently higher levels of insulin. (b) At 11 months, there was an interaction of diet with genotype on insulin tolerance over time (*p* < 0.05). Tg HFD mice showed increased glucose levels compared to Tg mice fed regular diet at all‐time points. Tg HFD mice had significant increase in glucose blood levels compared to Tg2576 mice fed regular diet at 6 months (30 and 60 min) and at 11 months (15, 30, and 60 min). (c) Total serum cholesterol was measured in 12.5‐month‐old mice. Cholesterol levels were higher in WT mice compared to Tg (*p* = 0.033) and in HFD compared to CTRL diet (*p* < 0.0001). The interaction of genotype by diet was not significant suggesting that the effect of the HFD on cholesterol levels was similar in Tg and WT mice. Post hoc analyses showed that the WT HFD (*n* = 12) and Tg HFD (*n* = 7) groups had significantly higher cholesterol levels than the WT (*n* = 11) and Tg (*n* = 7) mice on CTRL diet (*p* < 0.003 for the four comparisons). (d) FPLC chromatogram of the groups showed that HFD increased serum HDL cholesterol levels. NS, not significant

To measure insulin resistance, we applied the insulin tolerance test (ITT) at 6 and 11 months of age. At 6 months, HFD significantly increased the percentage of blood glucose level at all‐time points postinsulin injection in both WT and Tg mice in comparison with WT CTRL and Tg CTRL mice (Figure 1a). At 11 months, there was an interaction effect of genotype with HFD status on the percentage of change in blood glucose level over time (Figure 1b): In Tg mice (but not WT mice), HFD increased the percentage of blood glucose level above that of Tg CTRL mice at all‐time points postinsulin injection (*T*_15 min_: 122% vs. 96.7%, *T*_30 min_: 118% vs. 86.5%, *T*_60 min_: 112% vs. 76.7%) while these differences were not significant between the WT HFD and WT CTRL. HFD increased by approximately twofold the serum level of cholesterol in both WT and Tg mice groups (Figure 1c; 203 and 183 mg/dl, respectively) in comparison with WT CTRL and Tg CTRL (122 and 79 mg/dl, respectively). Interestingly and in accordance with the weight change between WT CTRL mice and Tg CTRL mice (Supporting Information Figure S2B), the cholesterol level of Tg CTRL mice was significantly lower than the cholesterol level in WT CTRL mice (Figure 1c). Lipoprotein separation on gel filtration column showed that the increased levels of cholesterol in mice treated with HFD were due to elevated HDL cholesterol levels in both WT and Tg mice (Figure 1d).

### 2.4 Dietary treatment differentially affected brain lipid classes' distribution

In the brain, lipid classes were affected by the dietary treatment in both WT and Tg genotypes. Tg mice showed higher neutral lipids content than WT under CTRL diet (1.29 ± 0.04 arbitrary units vs. 1.11 ± 0.03, Supporting Information Figure S3A). Total cholesterol was nominally higher in WT HFD (1.27 ± 0.11) than in WT CTRL (1.05 ± 0.03), but this did not reach statistical significance. Tg brain levels of total cholesterol under both diets were significantly higher than WT CTRL (1.21 ± 0.03 and 1.23 ± 0.04 for Tg CTRL and HFD, respectively, vs. 1.05 ± 0.03, Supporting Information Figure S3B). Cholesterol ester content relative to total cholesterol was significantly higher in Tg (53.22 ± 1.27) than in WT (48.53 ± 0.36) under CTRL diet and significantly higher in WT (51.82 ± 0.90) and Tg (57.17 ± 0.35) under HFD compared to their control diet counterparts (Supporting Information Figure S3C). Finally, free fatty acids (FFA), relative to total neutral lipids, were significantly higher in Tg (6.17 ± 0.41) than in WT (5.38 ± 0.26) under CTRL diet and significantly higher in WT (7.12 ± 0.67) and Tg (7.94 ± 0.48) under a HFD compared to their control diet counterparts (Supporting Information Figure S3D). These results indicate that there are intrinsic differences in brain lipid content between the two genotypes and that HFD alters the lipid milieu in the brain.

### 2.5 Tg mice fed with HFD showed improved spatial learning along with hypoactivity and anxiety‐like response

**Figure 2:**
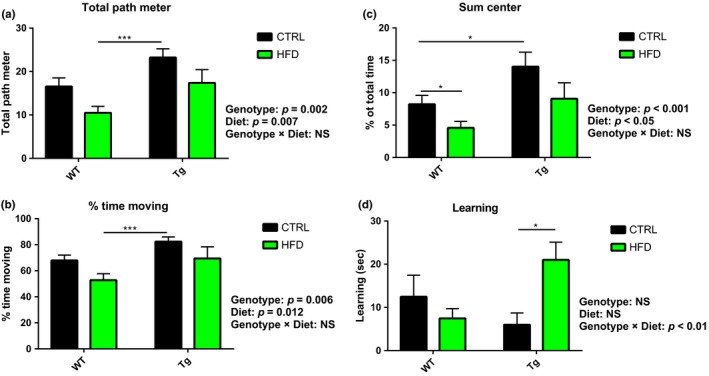
Effect of HFD and Tg2576 on exploration and learning in the open field and the Morris Water Maze tests. Behavior tests were performed at the age of 11 months to WT CTRL (*n* = 12), WT HFD (*n* = 16), Tg CTRL (*n* = 11), and Tg HFD mice (*N* = 7). The measured parameters are (a) total path in meters (b), percentage of time moving in the field, (c) percentage of total time spent in the center of the field, and (d) MWM test. Mean difference from first MWM day to last (fifth) day in number of seconds to reach the platform. Larger numbers reflect a larger difference, that is, better learning. NS, not statistics. *p < 0.05, **p < 0.01, ***p < 0.001

At age of 11 months, mice were submitted to behavioral tests, using open field test and MWM. Comparisons between the genotypes showed that Tg CTRL mice in comparison with WT CTRL covered significantly more total path meter (Figure 2a, 23.22 ± 6.2 vs. 17.13 ± 7.26 m, respectively), had higher percentage of time moving (Figure 2b, 82 ± 11.1 vs. 68 ± 15.6 m, respectively), and spent more time in the center of the field (Figure 2c, 14.03 ± 6.8 vs. 8.08 ± 5.2 m, respectively).

As expected due to the significant gain weight, in the parameter of total path meter (Figure 2a), the total path covered by Tg mice was significantly greater than that of WT mice, and HFD mice moved less than CTRL mice. Post hoc analyses showed that most of the effect was carried by the WT HFD mice (10.5 ± 5.8) having moved significantly less than Tg CTRL (23.2 ± 6.4); *p* < 0.0001). Figure 2b shows that the percentage of time moving in the open space test was greater in Tg compared to WT mice and in CTRL compared to HFD mice. Post hoc analyses showed that these effects were primarily due to significantly greater percentage of movement (*p* = 0.001) in Tg CTRL mice (82.4% ± 11.4) compared to WT HFD mice (52.8% ± 18.8). Similarly, Figure 2c shows that the percentage of time spent in the center of the open space platform was significantly longer in Tg compared to WT mice and in CTRL compared to HFD mice. Post hoc analyses showed that this effect was primarily due to significantly greater time spent in the center (*p* = 0.001) by the Tg CTRL mice (14.0% ± 7.0) compared to the WT HFD mice (4.6% ± 3.9). The interactions of genotype by diet were not significant for any of the open space test measures suggesting that the effect of HFD on the overall extent of movement and anxiety was similar in the Tg and WT groups. Finally, mice were tested for spatial learning. The learning rate in the MWM (time to reach the platform of the first minus the last day) did not differ by genotype nor by diet. However, the interaction of genotype by diet was significant, such that the differences between the learning rates in the Tg mice (for HFD, mean = improvement of 21.0 s ± 4.1 vs. CTRL 6.0 ± 2.7) were greater and in the opposite direction compared to WT mice (for HFD, mean improvement of 7.5 ± 2.3 vs. CTRL 12.5 ± 4.9). Post hoc analyses indicated a significant difference between Tg HFD and Tg CTRL (*p* = 0.037) groups.

### 2.6 HFD had no effect on Aβ_1‐42_ levels in the cortices of Tg mice

**Figure 3:**
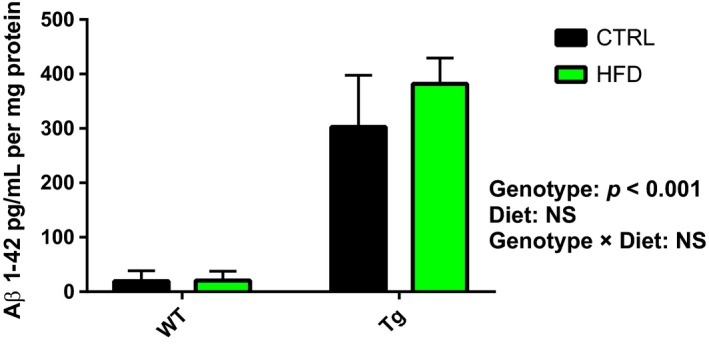
The effect of Tg and HFD on Aβ_1‐42_ levels in the cortex. ELISA test was used to analyze soluble and insoluble Aβ_1‐42_ fractions extracted from the cortex. The insoluble protein was diluted 1:180 with standard diluent. Tg mice fed regular diet (*n* = 6), Tg HFD mice (*n* = 5), WT HFD mice (*n* = 4), and WT mice fed regular diet (*n* = 3) were measured for Aβ_1‐42_ amount. Data are presented in bar graph of mean ± *SEM*

The signal transduction of insulin receptor in the brain is increased in insulin resistance which in turn is linked to increased brain levels of Aβ_1‐40_ and Aβ_1‐42_ (Dineley, Jahrling, & Denner, 2014). Thus, we investigated whether HFD affected the levels of Aβ_1‐42_ in the cortices of Tg mice. We found that Aβ_1‐42_ levels (302.9 ± 94.6, 381.7 ± 47.5, 19.4 ± 19.3, and 20.8 ± 16.8 pg/ml for Tg CTRL, Tg HFD, WT CTRL, and WT HFD, respectively) were significantly higher in Tg compared to WT mice, but there were no differences by diet and no interaction of genotype by diet, suggesting that HFD had no significant effect on Aβ_1‐42_ levels (Figure 3).

### 2.7 HFD restored AD genotype‐induced alterations in the transcription level of insulin receptor isoforms in the hippocampus

To investigate whether the effect of peripheral insulin resistance observed in HFD is reflected in the brain, we measured the transcription level of insulin receptor in the hippocampus. Insulin receptor (IR) has two isoforms expressed in the brain, A (IR‐a) and B (IR‐b). IR‐a is more prominent (Blazquez, Velazquez, Hurtado‐Carneiro, & Ruiz‐Albusac, 2014). When fed with HFD, IR‐a levels in Tg mice were found comparable to those of WT CTRL (Supporting Information Figure S4A). IR‐a levels were significantly lower in Tg compared to WT mice and in CTRL compared to HFD mice. Post hoc analyses showed that these effects were primarily due to a significant decrease in IR‐a levels (*p* < 0.01) in Tg CTRL mice (mean = 0.71 ± 0.06 relative arbitrary units) compared to WT HFD mice (1.18 ± 0.05), WT CTRL mice (0.96 ± 0.03), and to Tg HFD (1.04 ± 0.08). Similarly, for IR‐b, HFD normalized the transcription levels in Tg mice (0.78 ± 0.05 for CTRL and 1.05 ± 0.05 for HFD) which were similar to the IR‐b levels in WT CTRL (1.15 ± 0.16, Supporting Information Figure S4B). There were no differences in levels of hippocampal IR‐b expression in the WT mice groups (Supporting Information Figure S4B).

### 2.8 HFD improved BBB function and restored enlargement of the lateral ventricles volumes in Tg2576 mice

**Figure 4:**
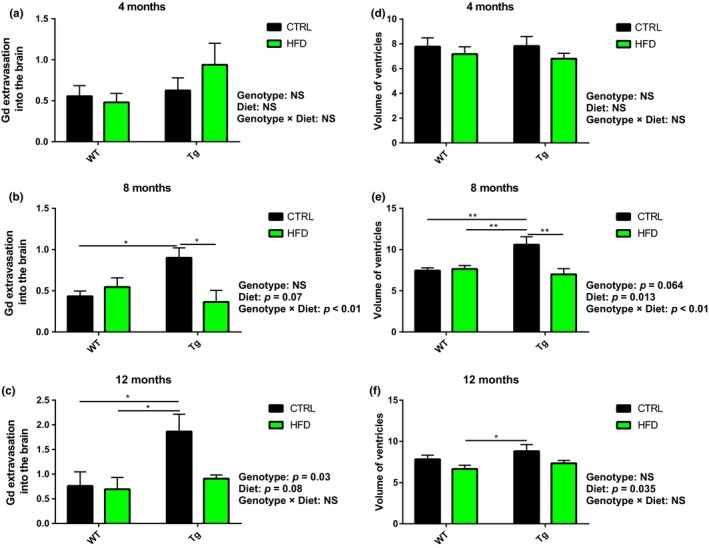
Tg genotype and HFD effects on BBB function and volumes of ventricles. MRI was performed at 4 (a, d), 8 (b, e), and 12 (c, f) months of age and measured contrast extravasations to the brain (a–c) and volume of the ventricles as calculated from the T2‐weighted MR images (d–f). (a) Graph bar of 4 months showing the mean slope representing contrast extravasation to the brain ± *SEM* in each mice group, *n* = 6–12. (b) Contrast extravasation at 8 months, *n* = 8–12. (c) Contrast extravasation at 12 months, *n* = 4–9. (d) Graph bar for 4 months shows the mean volumes of the ventricles ± *SEM* in each mice group, *n* = 14–19. (e) Graph bar for 8 months shows the mean volumes of the ventricles ± *SEM* in each mice group, *n* = 8–17. (f) Graph bar for 12 months shows the mean volumes of ventricles ± *SEM* in each mice group, *n* = 5–13. *p < 0.05, *p < 0.01. NS, not significant

We examined the role of HFD on BBB function using innovative MRI technology. We performed MRI scans at the age of 4, 8, and 12 months (Supporting Information Figure S5). BBB leakage was measured by the extent of contrast extravasation to the brain (Supporting Information Figure S6). At four months, there were no differences by genotype, diet, or an interaction of genotype (Figure 4a). At 8 months (Figure 4b), there was no difference by genotype, there was a trend level (*p* = 0.07) for less extravasation in the HFD diet (suggesting less BBB disruption), and there was a significant interaction of diet by genotype, such that the effect of HFD on the extent of extravasation was greater in Tg mice (for HFD mice mean extravasation = 0.36 ± 0.14, arbitrary units, and for CTRL mean = 0.9 ± 0.12) than in WT mice (for HFD mice mean extravasation = 0.55 ± 0.11 and for CTRL mean = 0.43 ± 0.06). Post hoc analyses showed a significant difference between Tg CTRL with Tg HFD (*p* = 0.011) and with WT CTRL (*p* = 0.021). At 12 months (Figure 4c), there was a significant effect of genotype on extent of extravasation with greater extravasation on Tg mice compared to WT. Similarly to the 8‐month analyses, there was a trend level (*p* = 0.08) for greater extravasation in the CTRL groups compared to the HFD group. Of note, although the interaction of genotype by diet did not reach significance (*p* = 0.13), possibly due to mice death and a resulting smaller N, the pattern was similar, that is, the effect of HFD on the extent of extravasation was greater in Tg mice (for HFD mice mean extravasation = 0.91 ± 0.07 and for CTRL mean = 1.87 ± 0.35) than in WT mice (for HFD mice mean extravasation = 0.69 ± 0.24 and for CTRL mean = 0.76 ± 0.29). Post hoc analyses showed significant differences between Tg CTRL with WT HFD (*p* = 0.04) and with WT CTRL (*p* = 0.050). Enlargement of ventricular volume is a known AD neuropathologic phenomenon. At 4 months, there were no differences by genotype, diet, or an interaction of genotype (Figure 4d). At 8 months (Figure 4e), there was a trend of difference by genotype (*p* = 0.064) with greater ventricles enlargement in the Tg mice, and a significant difference for diet, with a smaller ventricle volume in the HFD diet. Additionally, there was a significant interaction of diet by genotype, such that the effect of HFD on the ventricles volume was greater in Tg mice (for HFD mice mean volume = 6.99 µm^3^ ± 0.69 and for CTRL mean = 10.6 ± 0.96) than in WT mice (for HFD mice mean volume = 7.6 ± 0.43 and for CTRL mean = 7.5 ± 0.32). Post hoc analyses showed significant difference between Tg CTRL with Tg HFD (*p* < 0.01) and with WT mice on both diets. At 12 months (Figure 4f), there was no difference by genotype, and significant difference for decrease in volume in the HFD diet. Similarly to the Gd extravasation measurements, although the interaction of genotype by diet did not reach significance, the pattern showed beneficial effects for the HFD fed mice. In Tg HFD mice, mean volume was 7.35 ± 0.34 and for CTRL mean = 8.82 ± 0.8 while in WT HFD, the mean volume was 6.7 ± 0.46 and for WT CTRL mice, it was 7.8 ± 0.49. Post hoc analyses showed differences between Tg CTRL with WT HFD. Taken together, Tg CTRL mice had increased BBB leakage and larger ventricles volumes, compared to Tg HFD mice suggesting that HFD protected against BBB disruption and brain atrophy in AD‐like mice.

### 2.9 High cholesterol is associated with BBB integrity

Lastly, we examined whether the high levels of HDL cholesterol (Figure 1) were associated with BBB integrity. Indeed, higher levels of HDL cholesterol were associated with less BBB leakiness (*r* = −0.60, *p* < 0.01; Supporting Information Figure S7).

## 3 DISCUSSION

Initially, we hypothesized that the induction of insulin resistance‐like symptoms by HFD will aggravate AD‐like behavior and pathologies observed in Tg mice. We further postulated that BBB impairment might be a cause for this aggravation. These hypotheses were based on evidence showing that insulin resistance is linked with increased cognitive impairment (Ahtiluoto et al., 2010; Schnaider Beeri et al., 2004) and with the rationale that the association between insulin resistance and vascular disease (Semenkovich, 2006) could lead to vascular damage and to BBB abnormalities in the AD‐like mice model. To induce insulin resistance‐like phenotype, we fed WT and Tg mice with HFD (Surwit, Kuhn, Cochrane, McCubbin, & Feinglos, 1988) which, as expected, led to weight gain for both types of mice. The percentage change in the body weight between two months and 12 months was significantly larger in the Tg mice fed HFD and smaller in the Tg CTRL mice (Supporting Information Figure S2B) compared to the respective WT groups. HFD also induced insulin resistance at 6 and 11 months (Figure 1), which was significantly greater among Tg mice. These results are in line with other studies which reported increased weight in Tg HFD mice (Kohjima, Sun, & Chan, 2010) together with less weight gain in Tg CTRL mice due to hypothalamic leptin signaling dysfunction (Ishii, Wang, Racchumi, Dyke, & Iadecola, 2014).

Tg2576 mice are known to have impaired cognition and memory (Muthaiyah et al., 2014); however, the effect of HFD on cognition and memory is controversial and depends on the composition of the fat in the diet. Some experimental studies support the notion that HFD‐induced obesity exacerbates cerebral pathological alterations and the accompanying cognitive deficit in APP transgenic mice (Ho et al., 2004). In wild‐type mice, some studies suggest that HFD impairs cognition (Winocur & Greenwood, 2005) while other claim for no effect (Kesby et al., 2015) or even improvement of memory (Coscina et al., 1986) and protection from age‐related cognitive decline (Scheibye‐Knudsen et al., 2014). Thus, we next tested the effect of HFD on behavior. Mice were tested for locomotor activity and anxiety‐like behavior in the open field test and for learning in the MWM test (Figure 2). Tg CTRL mice demonstrated increased locomotor activity and decreased anxiety‐like behavior in comparison with WT CTRL mice. HFD induced a decrease in locomotor activity and increased anxiety‐like behavior in both Tg and WT mice. We cannot rule out the possibility that HFD‐weight gain diminished movement of mice to the center, rather than leading to anxiety per se. Our behavioral results are in agreement with other studies describing Tg mice as hyperactive animals that fail to habituate (Deacon, Koros, Bornemann, & Rawlins, 2009). Tg mice exhibit also disinhibitory tendencies which might explain their increased activity in the open field (Ognibene et al., 2005) suggesting that HFD “normalized” Tg mice motor and anxiety behaviors.

In our study, as expected, Tg CTRL mice performed poorly in the MWM and had worse learning compared to WT mice fed regular diet (Muthaiyah et al., 2014; Ognibene et al., 2005). However, Tg HFD mice showed significantly better learning than the Tg CTRL mice group (Figure 2d). The WT groups did not differ in their amount of learning suggesting that HFD may confer protection from cognitive impairment in the context of predisposition to AD.

The Tg groups did not differ in Aβ_1‐42_ levels in cortex. Our results are in accordance with a previous study showing that 4 months of western diet given to 3 and 12 months APPswe/PS1 mice did not affect aggregation of Aβ plaques in the cortex. Thus, the improved learning in Tg HFD mice was not due to decreased Aβ_1‐42_ levels (Figure 3).

Tg CTRL mice had significantly lower levels of transcription of insulin receptor in the hippocampus compared to all three other groups. HFD increased the level of insulin receptor in Tg mice back to the level of WT CTRL mice (Supporting Information Figure S4). It has been previously shown that elimination of the expression of insulin receptor using lentiviral vector in brain hippocampus of rats impaired their spatial learning (Grillo et al., 2015). Therefore, “normalized” insulin receptor expression in the Tg HFD group may underlie better learning in these mice.

Blood–brain barrier leakage in the total gray matter and cortex, measured by contrast‐enhanced MR imaging (van de Haar et al., 2016), has been recently reported in patients with MCI and early AD compared with control subjects. In this line, Tg CTRL mice developed BBB permeability in the cerebral cortex using albumin and Evans blue uptake (Ujiie, Dickstein, Carlow, & Jefferies, 2003). Our MRI methodology enables longitudinal monitoring of BBB leakage throughout the life of the mice, making it applicable for clinical purposes (Chassidim et al., 2013). We included two MRI‐based parameters: The lateral ventricles volume calculated from the T2‐weighted MR images and the BBB leakage based on the vessel function maps signal intensity plotted as a function of time. Tg CTRL mice demonstrated increased BBB leakage together with a significant increase in ventricular volume at 8 and 12 months in comparison with all other mice groups. Contrary to our primary hypothesis, our data suggest that in AD‐like mice, HFD protected the brain by decreasing BBB permeability to the contrast agent together with a substantial decrease in the volume of the lateral ventricles suggesting less brain atrophy (Figure 4). Due to MRI resolution constraints, we cannot rule out the possibility that the extended penetration of Gd is due to Blood‐CSF barrier leakage rather than BBB damage or a combination of both.

The CTRL diet and the HFD used in the current study differ from each other not only in the percentages of fat but also in the composition of the fatty acids. According to Supporting Information Table S1, the HFD is low in carbohydrates and high in monounsaturated fatty acids in comparison with the regular diet. The ability of high‐fat diets to affect the BBB is controversial. On the one hand, 4 months of western diet given to 3 and 12 months APPswe/PS1 mice did not compromise BBB integrity (Theriault, ElAli, & Rivest, 2016). However, in another study, HFD‐induced obesity was shown to exacerbate BBB permeability in aged mice (24 months) measured by IgG extravagated to the hippocampus (Tucsek et al., 2014). The percentage of saturated fat in the diet of the latter study was the same as in our study. However, our HFD contained more monounsaturated fatty acids (48% vs. 34% in the Tucsek et al. study) and less polyunsaturated fatty acids (16% vs. 32%) suggesting that in the context of AD, monounsaturated fatty acids may protect the brain despite weight gain and peripheral insulin resistance. This is consistent with evidence suggesting that monounsaturated fatty acids prevent the deleterious effects of obesity on locomotion and brain activity (Sartorius et al., 2012) and are associated with decreased risk of AD (Morris et al., 2003).

Our analyses of brain lipid content (Supporting Information Figure S3) seem to indicate that brain neutral lipids content, including free and esterified cholesterol as well as FFA, is higher in Tg than in WT mice. Significant associations were recently observed between the abundance of unsaturated fatty acid (UFAs) with domain‐specific cognitive performance assessed during life (Snowden et al., 2017) suggesting that UFAs metabolism is significantly dysregulated in the brains of patients with varying degrees of AD pathology. Moreover, the toxic process of Aβ formation and aggregation occurs preferentially in lipid rafts; therefore, alterations in the lipids milieu of different cells in the brain might influence this process as well (Morgado & Garvey, 2015). Our results show that both genotypes are susceptible to an increase in brain lipids under a HFD regime. In particular, FFA and cholesterol esters proportions increase. This may be interpreted as either an increased uptake of both lipid classes or rather, as an increase in the esterification process due to an initial increase in the FFA component.

Ventricular enlargement is a highly reproducible measure of Alzheimer's progression, owing to the high contrast between the CSF and the surrounding brain tissue on T1‐weighted images (Frisoni, Fox, Jack, Scheltens, & Thompson, 2010). The ventricles expand by 5%–16% per year in patients with AD and by 1.5%–3.0% per year in healthy elderly individuals. The relatively low error variance gives this measure excellent power to detect consistent changes over short follow‐up intervals (for example, 6 months; van de Pol et al., 2007). Therefore, our results showing that Tg HFD mice had less enlargement of the ventricles might have important future clinical implication.

High‐fat diet induced increased levels of HDL cholesterol serum at 12 months for both mice genotype, while Tg CTRL mice had the lowest level of cholesterol (Figure 1). HDL has anti‐elastase activity which may represent a protective effect on the BBB in pathological conditions, involving neutrophil activation and subsequent elastase release (Ortiz‐Munoz et al., 2009). In particular, HDL may be able to transport α1‐antitrypsin into the cells where it could thwart the deleterious effects of intracellular elastase on the BBB (Houghton et al., 2010). Because HDLs display antioxidant effects (Barter et al., 2004) and reconstituted HDLs were shown to restore endothelial function in hyperglycemic conditions (Nieuwdorp et al., 2008), it is possible that they may have beneficial effects on the BBB in these pathological conditions. Indeed, higher serum HDL was associated with lower levels of BBB injury in multiple sclerosis patients (Fellows et al., 2015). Similarly, our results show that high HDL levels are significantly associated with less BBB leakiness (Supporting Information Figure S7) consistent with the prospect of HDL‐induced BBB protection.

Our study has limitations. Tg2576 mice develop little atherosclerosis and thus this study should be repeated in animals such as APOE knockout mice to assess the role of our HFD, which is rich in monounsaturated fatty acids and which increases substantially HDL cholesterol, on cerebrovascular disease. It is possible that the multiple MRI assessment caused stress which might have affected the behavioral tests. For example, chronic stress has been shown to exacerbate neurodegeneration and cognitive impairment through a corticotropin‐releasing factor receptor‐dependent mechanism in Tg2576 mice (Carroll et al., 2011). We also tried to validate the BBB results obtained with the MRI using measurements of tight junction levels (using mRNA and western blot, data not shown) but failed to see any differences between the groups. This is most likely because we used whole brain lysates which are diluted in tight junction‐related genes in comparison with purified brain capillaries.

In summary, our results suggest a protective effect induced by HFD on AD‐like mice, through mechanisms that involve better BBB properties and brain morphology (normal ventricle volume, consistent with less brain atrophy) as well as higher insulin receptor RNA expression and higher HDL cholesterol but without reduction in Aβ_1‐42_ parenchymal load.
