# Supplementary figures and images for: High‐fat diet protects the blood–brain barrier in an Alzheimer's disease mouse model
Source: Aging Cell. 2018 Aug 6;17(5):e12818. doi: 10.1111/acel.12818 (PMC6156545; doi:10.1111/acel.12818)

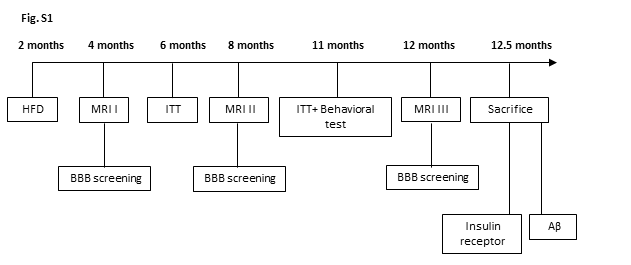

Supplement: Supplementary file 1 [file ACEL-17-e12818-s001.tif]

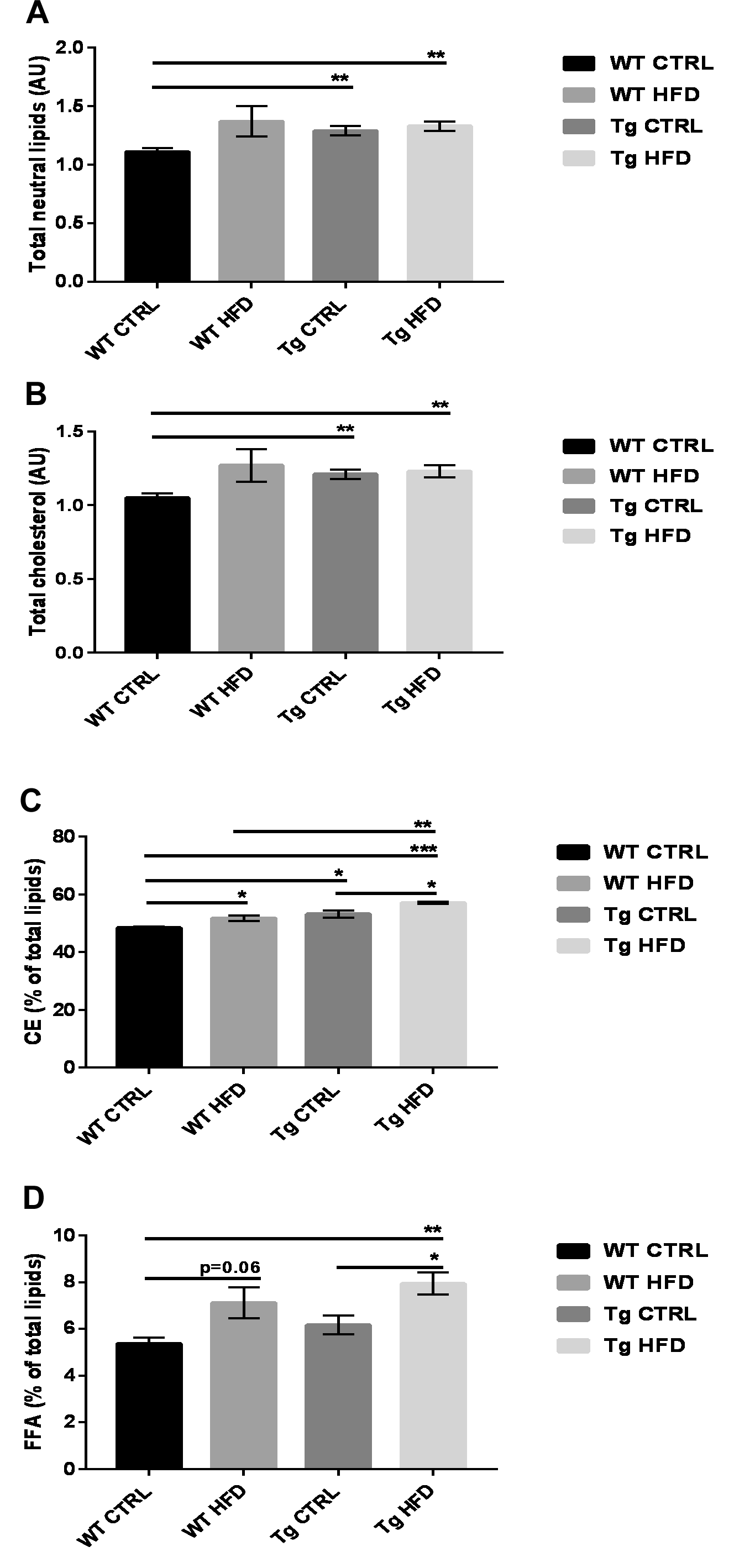

Supplement: Supplementary file 3 [file ACEL-17-e12818-s003.tif]

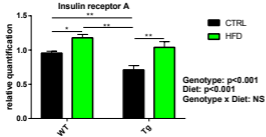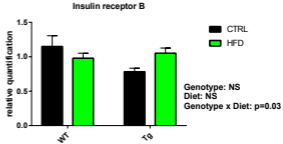

Supplement: Supplementary file 4 [file ACEL-17-e12818-s004.pdf]

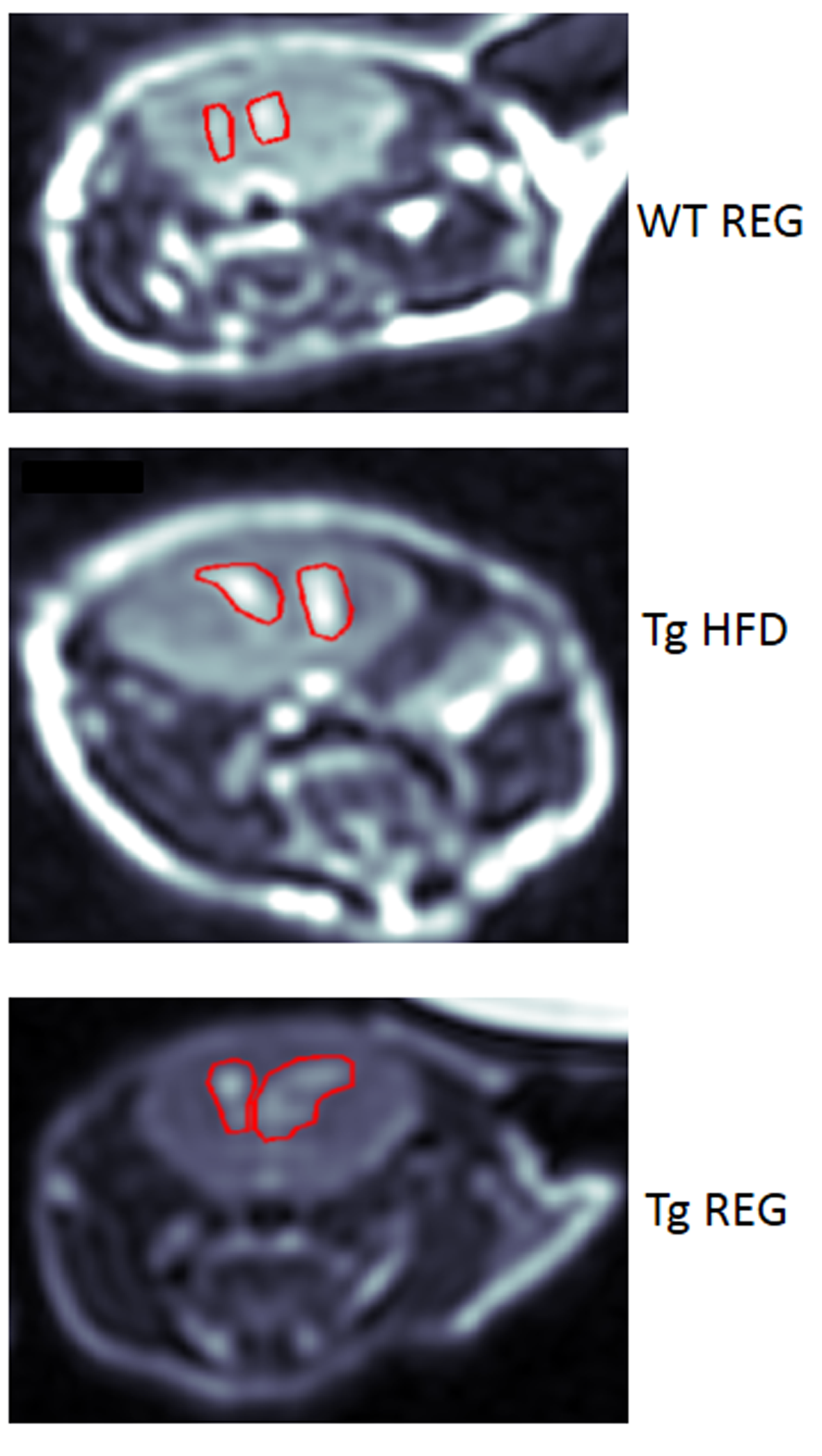

Supplement: Supplementary file 5 [file ACEL-17-e12818-s005.tif]

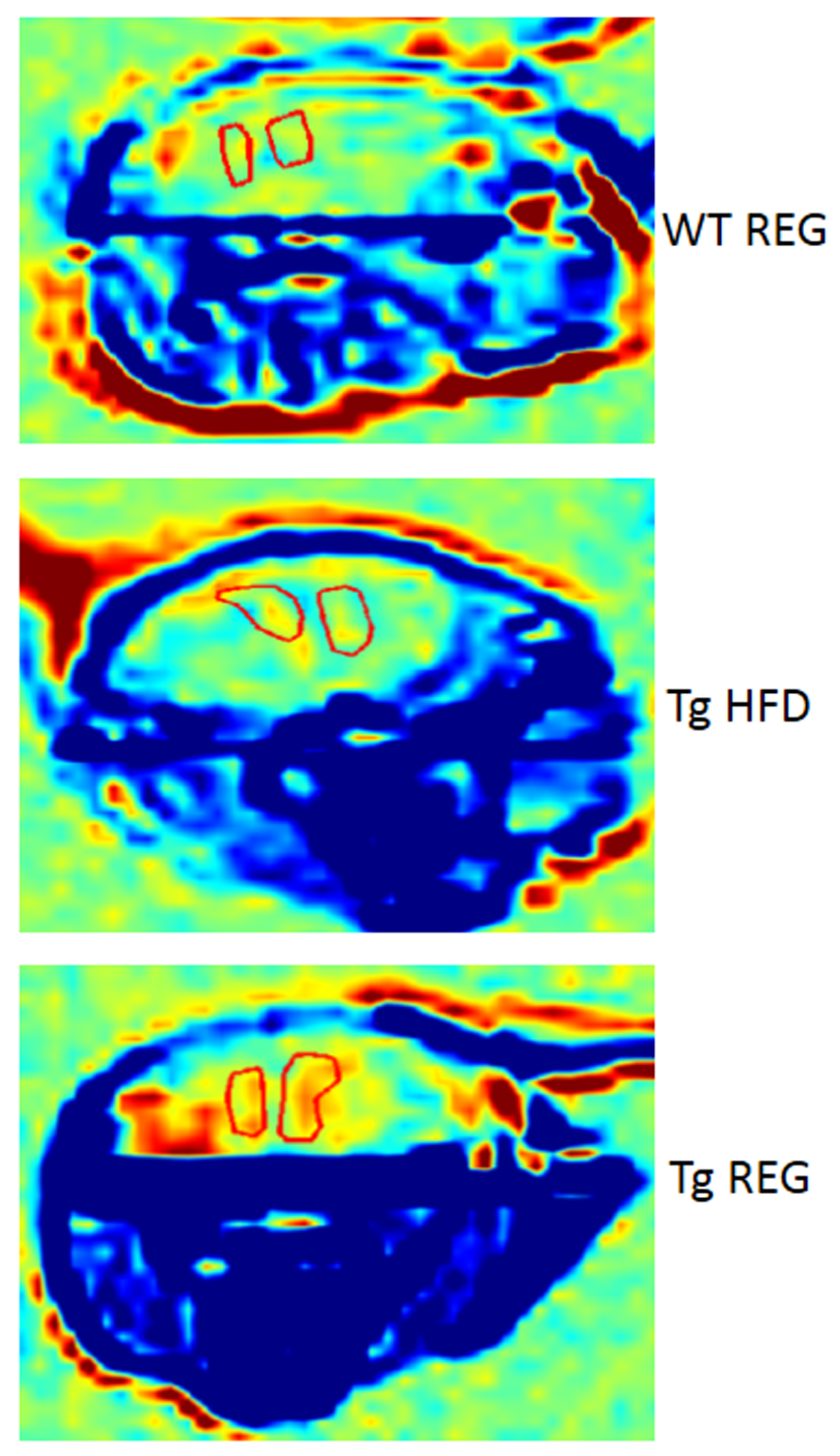

Supplement: Supplementary file 6 [file ACEL-17-e12818-s006.tif]

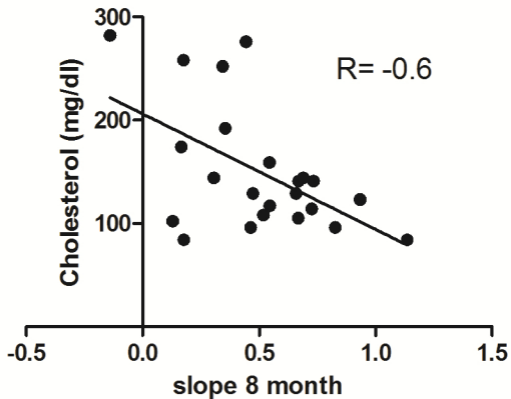

Supplement: Supplementary file 7 [file ACEL-17-e12818-s007.pdf]
