# Supplementary material for: High‐fat diet protects the blood–brain barrier in an Alzheimer's disease mouse model
Source: Aging Cell. 2018 Aug 6;17(5):e12818. doi: 10.1111/acel.12818 (PMC6156545; doi:10.1111/acel.12818)
Supplement: Supplementary file 8 [file ACEL-17-e12818-s008.pdf]

		HFD	Regular
	**Unit**	**TD.06414**	**2018S**
**Protein**	% by weight	23.5	18.6
	% kcal	**18.3**	**24**
	source	Casein	soybean meal, corn gluten meal, wheat
**Carbohydrate**	% by weight	27.3	44.2
	% kcal	**21.4**	**58**
	source	sucrose, maltodextrin	corn, wheat
**Fiber**	% by weight	6.5	*14.7
	% kcal	N/A	N/A
	source	cellulose	wheat, corn, soybean meal
**Fat**	% by weight	34.3	6.2
	% kcal	**60**	**18**
	source	Lard, soybean oil	Soybean oil (added); oils inherent in grain ingredients
