# Supplementary material for: High‐fat diet protects the blood–brain barrier in an Alzheimer's disease mouse model
Source: Aging Cell. 2018 Aug 6;17(5):e12818. doi: 10.1111/acel.12818 (PMC6156545; doi:10.1111/acel.12818)
Supplement: Supplementary file 9 [file ACEL-17-e12818-s009.docx]

**Methods**

**Mice and Diet**

C57Bl/6J wild-type (WT) and Tg2576 male mice were used. The Tg2576 mice express 695 amino acid form of human *APP* with the 'Swedish' mutation, containing the K670N/M671L FAD mutation, placed under the transcriptional control of the hamster prion gene promoter. Starting at 2 months of age, mice were randomly assigned for control diet or HFD (See Table [1](https://nutritionandmetabolism.biomedcentral.com/articles/10.1186/1743-7075-9-58#Tab1) for composition of the diets). The HFD food was kept at -20ºC and replaced every two days to avoid fat oxidation. The study had a 2 by 2 design (Tg2576 vs. control mice; HFD vs. regular diet). For convenience, the following abbreviations were given: WT mice fed control diet – 'WT CTRL', WT mice fed HFD - 'WT HFD', Tg2576 mice fed control diet - 'Tg CTRL' and Tg2576 mice fed HFD are 'Tg HFD'. Mice were placed each in separated cages, and their weight was recorded monthly. Insulin Tolerance Test (ITT) was performed at 6 and 11 months of age. At 11 months mice were tested for memory and learning. At 4, 8 and 12 months they were assessed for BBB disruption using MRI and serum was collected. Mice were sacrificed at 12 months and brain parenchyma was dissected for rtPCR and Aβ measurements. All institutional and national guidelines for the care and use of laboratory animals were followed.

**ITT tests**

Mice fasted for two hours. Each mouse was weighted and the insulin dose for the injection was calculated according to 0.75 U/kg body weight. Insulin was prepared at 0.075 U/ml (7.5µl of 100U/ml insulin in 10ml PBS). Blood glucose was measured before ITT to assess the baseline value (time 0). Blood glucose level was determined by glucometer on a blood sample driven from the tail by snipping. At time 0, insulin was injected intraperitoneally (i.p) according to the calculation of body weight (g) X 10 µl of 0.075U/ml insulin solution. Blood glucose was measured at 15, 30 and 60 minutes following insulin injection.

**RNA isolation and real-time PCR analysis of Insulin receptor**

Total RNA was extracted from brain tissue using NucleoSpin kit (Macher-Nagel, USA), according to the manufacturer's instructions. Total RNA was dissolved in nuclease-free water and stored at -80ºC. Reverse transcription of 2µg cDNA was performed using high capacity cDNA RT kit (Applied Biosystems, Thermo Fisher Scientific, USA). Primers were purchased from Hy-labs, Israel. The sequences are as follows: For Insulin receptor isoform A- 5'-3': FW- TTTTTGTCCCCAGGCCATCCCG; RE- GTGTGAGTGTGGTGGCTGTC. For Insulin receptor isoform B- 5'-3': FW- AACCTCTTCAGGCAATGGTG; RE- GGGGAAATCTGGAAGTGTGA.

The set of NONO primers was used as internal control for each specific gene amplification. The relative levels of expression were quantified and analyzed using step one plus (Applied Biosystems, Thermo Fisher Scientific, USA). Samples were performed in triplicates. The real-time value for each sample was averaged and compared using the CT method, where the amount of target RNA (2^-ΔΔCT^) was normalized to the endogenous NONO reference (ΔCT) and related to the amount of target gene in tissue cells, which was set as the calibrator at 1.0.

**Measurement of cortical Aβ**

For quantitative assessment of cortex formation of Aβ peptides, frozen tissue was extracted in a two-step extraction. Tissue was homogenized in 1% Triton X-100, diluted in 25mM phosphate-buffered saline containing 137mM NaCl and protease inhibitors cocktail (Roche Biochemicals, Indianapolis, IN, USA) and centrifuged for 1 h at 4°C at 100000g. The fraction was designated Aβ_sol._ The remaining pellet was then sonicated in 5M guanidine HCL with 50mM TRIS PH=8 and protease inhibitors cocktail, incubated for 2 h at 25°C, and centrifuged at 13000g for 20 min at 4°C. The latter fraction was designated as Aβ_insol_. Aβ1-42 was measured by sandwich ELISA (WAKO, Osaka, Japan) according to manufacturer instructions with dilution of 1:180.

**Open field test**

The open field apparatus is a square field (50 × 50 × 30 cm) made of white acrylic material. The mouse was placed in the corner of the open field at the beginning of the test and allowed to move freely for 5 min. The floor and walls of the field were cleaned thoroughly with 70% ethanol and air-dried after each trial to remove olfactory cues. Trials were recorded using a ceiling-mounted video camera (Tracker VP200; HVS Image, Hampton, England) and analyzed off line. The image of the field was divided into 16 equal squares (4 inner and 12 outer). The total distance (= total path), the percent of time moving and percent of cells used were evaluated as an index of locomotor activity, and the total center time (= percent time spent in the center 4 inner cells) was evaluated as an index of anxiety. The data analysis was performed using HVS image field 2100 software.

**Morris water maze (MWM)**

The spatial reference learning and memory of all mice were assessed using the MWM. Mice were tested in a 140-cm circular pool filled with water at 25–27 °C and made opaque by the addition of nontoxic white acrylic paint. In a pre-learning phase, mice were trained to mount a submerged escape platform (20′20 cm) in a restricted region of the pool (contained by a circular vinyl insert 60 cm in diameter). For the subsequent trials, the pool was divided into four quadrants. The four start positions were located at the intersections of the quadrants. A platform was hidden 1.5 cm below the water level. The mice were first placed into the water facing the wall of the pool and allowed to swim until it located and climbed onto the submerged platform. Each mouse received four trials per day for six consecutive days of the learning phase. The start position was changed in each trial. The swim time to reach the platform was recorded in each trial. Mice failing to reach the platform in 60 s were led to the platform and placed on the platform for 15 s. On the 7th day, in the probe trial, the platform was removed from the pool and mice were allowed to search for the platform for 60 s. All trials were monitored by a camera above the pool. The time spent searching for the platform and the target quadrant frequency were recorded. Prior to each entry into the water maze pool, mice were placed in a thermoregulated holding chamber filled with water (3 cm deep) for 90 s, so that anxiety responses (related to experimenter handling and exposure to water) could be minimized. To control for potential hypothermia, following each trial, mice were towel‐dried and returned to a prewarmed home cage. Water maze activities were monitored with the HVS image 2100 Plus Track video tracking system (Buckingham, UK). Mice were tested during the second and third hours of the night portion of the light cycle. All animals were individually coded, and investigators were blind to group designations throughout testing.

**MRI evaluation of BBB disruption**

MRI was performed using a 1.5T GE system with a standard phased array wrist coil. Mice were anesthetized with ketamine and xylazine for 1 hour and an I.V. Cannula (391349, BD, USA) was placed in their tail vein for injection of a standard dose (0.2 ml/kg) of the contrast agent Gd-DTPA (MW 550, DOTAREM, 0.5mmol/ml, Guerbet, France). MRI included pre- and post-contrast spin echo T1-weighted MRI as well as fast spin echo T2-weighted MRI sequences. T1-weighted MRI was continuously acquired up to 25 min after contrast injection. For the analysis of the MR images we exploited a novel methodology based on delayed contrast extravasation MRI for calculating vessel function maps (VFMs) of the brain with high resolution and high sensitivity to subtle BBB disruption ([Zach *et al.* 2015](#_ENREF_44)). VFMs are calculated by subtracting the late images from the images acquired immediately after contrast injection in an attempt to depict local BBB disruption. The ventricles volume was found to differ between the Tg2576 regular fed diet and the other groups, thus regions of interest (ROIs) were plotted around the ventricles on the T2-weighted MR images. These ROIs were than copied to the co-registered T1-weighted MR images and the average signal intensity was calculated and plotted as a function of time. Finally, the slope of the signal variation was evaluated and compared between the four groups. Exclusion criteria were mice with only one ventricle detected in the MR images (due to suboptimal positioning) and mice that moved during scanning (resulting in low quality images).

**Lipid measurements in serum**

Colorimetric enzymatic procedures were used to measure serum total cholesterol (Chol, Roche/Hitachi, Roche Diagnostics). Mouse serum was defrosted a day before the experiment, and 30µl aliquot were loaded in a special tube and diluted with 60µl of PBS. The Cobas Mira autoanalyzer (Roche) was used for lipid detection of serum samples. Measurements of serum cholesterol and lipoproteins were determined by high-performance liquid chromatography.

**Lipid extraction and analysis in brains**

Samples for lipid extraction and analysis were prepared from frozen brain tissues that were left after the dissection of cortex, striatum, hippocampus, hypothalamus, amygdala and cerebellum. Samples were weighted and homogenized with saline in plastic tubes on ice at concentration of 1mg/12.5µl. 250µl from each homogenate were utilized for lipid extraction and analysis with thin-layer chromatography (TLC). The total lipid amount was calculated after aliquot evaporation to constant weight and the lipid species distribution was analyzed by TLC applying 150 µg aliquots. Samples were reconstituted in 10 µL of Folch mixture and spotted on Silica-G TLC plates. Standards for each fraction were purchased from Sigma Aldrich (Rehovot, Israel) and were spotted in separate TLC lanes i.e., 50 µg of triacylglycerides (TG), cholesterol (C), cholesteryl esters (CE) and free fatty acids (FFA). Plates were then placed in a 20x20 cm TLC chamber containing petroleum ether, ethyl ether, and acetic acid (80:20:1, v/v/v) and run for 45 min. TG, C, CE and FFA bands were visualized with Iodine, scanned and quantified (Epson V700).

**Statistical analysis**

All values are expressed as mean ± SEM. For the weight and the ITT measurements a Two-way mixed design ANOVA test was applied with repeated measures. Bonferroni post hoc test was used to show differences between the groups. Two-way ANOVA and Tuckey post hoc tests were used for the MWM, open field test, MRI analysis, expression level of the insulin receptor RNA, serum cholesterol and brain lipid levels. For simplicity and consistency purposes, all results are presented as main effect of genotype, then main effect of diet, and lastly the interaction of genotype with diet. If any of these was significant, we then present the differences that were significant per posthoc analyses.

SPSS version 21.0 (IBM corporation, NY, USA) was used for data analysis.
